# Contextual, structural, and mental health experiences of children of women engaged in high-risk sexual behaviour in Kampala: a mixed method study

**DOI:** 10.3389/fpubh.2023.1185339

**Published:** 2023-12-15

**Authors:** Agnes Ssali, Georgina Nabaggala, Michael C. Mubiru, Ibrahim Semakula, Janet Seeley, Rachel King

**Affiliations:** ^1^London School of Hygiene and Tropical Medicine Uganda Research Unit, Medical Research Council, Uganda Virus Research Institute, Entebbe, Uganda; ^2^Department of Global Health and Development, London School of Hygiene and Tropical Medicine, London, United Kingdom; ^3^Department of Epidemiology and Biostatistics, University of California, San Francisco, San Francisco, CA, United States; ^4^INSERM, Montpellier, France

**Keywords:** children, experiences, sex worker, mothers, livelihoods, contextual, structural, mental health

## Abstract

Children born to women who sell sex for money or commodities may face economic and social insecurity because of their mother’s work, particularly in settings where sex work is illegal. From October 2020 to May 2021, we conducted a study with 60 children aged 12–24 years, born to sex workers in Kampala, Uganda. The children took part in 60 semi-structured interviews, 20 life history interviews, and 4 focus group discussions, which were used to explore their social, economic, and mental health experiences and investigate their vulnerabilities and resilience. Quantitative data were collected using REDcap, and descriptive analysis was done using Stata 14. Qualitative data were collected using semi-structured topic guides, and data analysed thematically. We explored findings in relation to a wellbeing framework. The findings showed that children experienced contextual and structural hardships, including incomplete and irregular schooling, a lack of privacy at home, food insecurity, and physical and psychological violence from relatives and sometimes from their mothers. Some children reported mental wellbeing struggles with hopelessness, nervousness, and sadness. Alcohol and drug use were common in most families. Community social network support systems, including neighbours and grandparents, were important; most children had absentee fathers. Some children suspected or knew how their mother earned her income. Resilience for most children was tagged to support from close networks and financial support from the government and civil society. Children of sex workers in Kampala experience structural, contextual, and mental health challenges but have a positive attitude towards the future. It is important to strengthen community support systems for these children and those living in similar circumstances in low- and middle-income countries.

## Introduction

Women who provide sex for money and commodities are often mothers, and they must manage parenting and their maternal identity in addition to their work identities (1–3). Such women may live in impoverished situations as they strive to support their children (3–6).

Children of female sex workers (FSW) are seldom considered to be a distinct at-risk population. However, they may face the same social, economic, and health challenges as their mothers (7, 8), especially where sex work is illegal, which is the case in many countries in sub-Saharan Africa, including Uganda. The women who offer sexual services in such settings face harassment and sometimes arrest.

In Uganda, the children’s mothers, conscious of the risks posed by their work environment, may opt to send their children to be raised by relatives in rural areas, placing them in environments deemed safer for socialisation (3, 9). However, this option may not be available to all women, or they may prefer to keep their children with them. The children who stay with their mothers may be left without care and support overnight while their mothers work or for longer periods when their mothers are detained by the authorities (7, 10). Childcare options may be limited unless the women arrange the support amongst themselves since childcare services may be lacking or unaffordable in their communities (11).

The literature on the children of sex workers indicates that children may themselves become involved in risky behaviours, including risky sex, alcohol, and substance use, that may increase their vulnerability to HIV and other sexually transmitted infections (7, 12, 13). A study of adolescents in Kenya indicated that poverty was a key driver of such behaviours connected to sexual experimentation and transactional sex (14).

In this study, focusing on Kampala, Uganda, we investigated the socio-economic and health context that children of women who sell sex grow up in, with the aim of identifying areas for possible low-cost, culturally relevant, feasible, acceptable, and accessible interventions to support these families.

We adopted a wellbeing framework developed by Sarah White (15), which centres wellbeing on the person and their priorities and perspectives. It emphasises strength rather than needs and highlights people’s own experiences and perceptions of life. The framework uses three dimensions: material/financial, human (competencies, psychological, among others), and social/relational wellbeing. We consider wellbeing as a process where the three main dimensions are interconnected within context/space and time.

## Materials and methods

This was a pilot, descriptive, cross-sectional study using both qualitative and quantitative data collection and analysis methods.

### Study setting

We conducted the study among the children of a cohort of women at high risk of HIV, including female sex workers, some of whom were living with HIV and were attending a specialised clinic in Kampala for at least 2 years. Sex workers in this study were women who reported that they engaged in high-risk sexual behaviour for money and commodities. It was an inclusion criterion to join the Good Health for Women (GHWP) cohort.

The GHWP clinic offered health services, including HIV treatment, counselling, syndromic management of sexually transmitted infections, and HIV prevention support through peers and community outreach to women at high risk of HIV and their regular sexual partners and children aged below 5 years. The clinic was located in the central division of Kampala city and accessed by women at high risk of HIV from surrounding areas and a nearby district of Wakiso.

### Sampling and recruitment

At the time of the conduct of this study, we targeted approximately 2,000 women who had attended the clinic at least once in the past year and had reported having children. Engagement in sex work was confirmed from the existing database and peer support system. To identify participants for the study, we looked for women in the database who reported having children aged 12–24 years and living in the same household (inclusion criteria). We then targeted those children for the study. We found a list of 200 women who fit the inclusion criteria. Most of these women had shared their phone contacts with GHWP, and a majority could be contacted by phone. One-third did not have phones and were contacted through their peer leaders who knew which women had children aged 12–24 and were living with them in the same household.

During the phone call, the research team explained the purpose of the study to the participants. They asked the women to confirm whether they had children aged 12–24 years and also enquired about the gender of the children. The women were asked about their willingness to participate in the study together with one child of their choice. They were free to choose any child of theirs aged 12–24 years regardless of their gender but living with them in the same household. The study had more girls than boys who had been chosen by their mothers in the targeted age group of 12–24 years. When selecting the mother and child pair for the study, we excluded women whose children were at boarding schools, residing outside Kampala or were below 12 years old.

At the clinic, women were screened to check their names, phone contacts, and identification. Children were separately requested to ascertain their relationship with the accompanying woman, and their consent to take part in the study was sought. Two women had to be excluded from the study because the children revealed that they were not their biological mothers.

### Data collection

Data were collected between October 2020 and May 2021. The questions in the questionnaire were adapted from a Uganda Ministry of Gender Labour and Social Development form that was used for assessing the social wellbeing of orphans and vulnerable children in communities. The children selected for the study were asked to respond to a questionnaire which had 32 semi-structured questions on the socio-demographic characteristics, age, schooling, social services in the community, use of drugs observed in the community, their food and shelter experience at home, and any form of violence they had experienced. The questionnaire-based interviews took 30 min on average (see Supplementary material for the questionnaire).

Collected data were uploaded to the Unit RedCap server daily. Data were checked for consistencies, outliers, and completeness and then exported to STATA V.14 (Stata Corp, College Station, TX, United States) for further data cleaning, management, and analysis. Data collected included socio-demographics and the children’s experiences of vulnerability and resilience under the following themes: social relationships, financial wellbeing, human wellbeing (quality of the environment and personal safety and emotional health), and self-reported assessment level of anxiety and depression, where the primary outcome was the level of anxiety and depression.

We used a structured 10-point checklist for anxiety and depression (KQ10 anxiety and depression checklist)^1^ to assess the mental health status of the women and the children. The KQ 10 checklist had 10 questions that asked the respondents to rate how they had been feeling in the past 4 weeks. We used this to gauge the mental health of the children. The aim was not to diagnose mental health illness but only to obtain an assessment of the respondent’s mental health and wellbeing. It was used as a tool to gauge what the children felt. Questions included how often they felt tired, nervous, hopeless, or restless, among others. This tool was adopted and used as a starting point to understand the children’s feelings. The tool had been used before to gauge mental wellbeing, but not among this study population.

The interviews were conducted at the clinic premises, which were at the time closed to services and therefore presented a quiet, secure environment. We did not conduct any individual analysis in this study.

All available children were contacted to take part in the focus group discussions (FGDs) to complement the quantitative descriptive data. The FGD guide had several topics for discussion, which included community activities, socialisation, child upbringing, use of alcohol and drugs, HIV and AIDS, and support systems and networks in the community (please see Supplementary material for more details).

We conducted four FGDs: two with boys, one for the group aged 12–17 years and the other for the group aged 18–24 years; and similarly, two with girls, one for the group aged 12–17 years and the second for the group aged 18–24 years. We had 10 participants in each group. The FGDs were also conducted at the clinic premises. The FGDs took between 50 and 90 min.

After the quantitative interviews and FGDs, we purposively selected a sample of 20 children across different age groups to take part in life history interviews. The life history interviews explored the experiences of the children along a continuum from when the children understood what was happening in their environment until the present. Individual life histories took between 40 and 60 min. There were no repeat interviews. The children shared their lived experiences. Five children reported domestic violence in the questionnaire-based interviews; this was reported in the quantitative findings. During the life history interview, all the children were asked about violence. All data were anonymised.

The life histories were conducted using a semi-structured topic guide (see Supplementary material) at the child’s home or in a convenient place near their home.

### Data management and analysis

Questionnaire data was collected by experienced research assistants using encrypted tablets linked to the Redcap data management programme. A backup of the data was done every day on the MRC/UVRI and LSHTM Uganda Research Unit server.

Quantitative data were transferred and coded into Stata14. Statistical analysis was performed using STATA V.14 (Stata Corp, College Station, TX, United States). All variables were checked for missing data and inconsistencies. Distribution of the data using summary measures and graphical displays was examined to identify outliers and verified against source documentation. The participants’ characteristics were summarised using means ±SD or medians (IQR) and percentages. Categorical variables were reported as numbers and percentages. The chi-square test (χ^2^) was used to determine differences in age categories of mothers and children and baseline characteristics of both. Based on the χ^2^-test, variables that were significantly associated (*p* < 005) with the different age categories were used in the subsequent analysis.

Bivariate logistic regression analysis was used to determine the self-reported assessment level of anxiety and depression. Based on the results obtained from the bivariate logistic regression models, variables that were found to have an independent association were: feeling hopeless, feeling nervous, feeling sad, feeling worthless, and feeling restless.

These were added to the final multivariate logistic regression model adjusting for age-based *a priori* knowledge of a known confounding effect of age on the outcome. From the unadjusted and adjusted models, value of ps, OR, and corresponding 95% CIs were reported.

The life history interviews and FGDs were audio-recorded. The research assistants transferred the data to a password-protected computer and deleted the recording from the voice recorder. Qualitative data were transcribed and translated from the vernacular language (Luganda) to English. The first author discussed three life history transcripts and two focus group discussion transcripts with the research assistants, and they agreed on the main themes in the data and chose corresponding codes. The team read through the scripts, agreed on the codes, developed a code book, and then coded all the interview scripts. After coding, responses were grouped to see the patterns from the data following the framework. The data coding for the 20 life histories was managed using Nvivo12, a qualitative electronic data analysis package. The focus group discussions were manually coded. Manual coding, in this case, refers to reading and coding scripts without the use of an electronic data analysis package.

The data were analysed for patterns using the inductive and deductive approaches of analysis, and later, the team agreed on overarching themes and subthemes. The themes in the paper were mapped onto the wellbeing framework. These data were not individualised but were collated in the framework using quotes from the children without indicating the age of the individual children to avoid identification; instead, we provided the broad age group.

### Ethical considerations

This study was approved by the Uganda Virus Research Institute Research Ethics Committee and the Uganda National Council for Science and Technology. The study was also approved by the University of California, San Francisco Human Research Protection Programme Institutional Review Board (IRB) and the London School of Hygiene and Tropical Medicine ethics committee.

All the children gave their own written consent/assent before they were interviewed. Children aged 18 and above were asked for consent and assent for those aged below 18 years. Consent and assent were obtained from the children individually without the presence of their mothers.

## Results

The findings from this study have been presented using descriptive data from the quantitative data and material, and we have included select quotes from the children in the qualitative findings.

We have shared the demographic characteristics of the mothers and the children. The mother’s data are shared to provide information on the children’s background. We have presented the qualitative data from the children around three main themes. These include the social relationships of the children, their material and financial wellbeing, and their human dimensions, including their health and mental wellbeing. We adopted the wellbeing framework as we shared these themes, taking into consideration the objective and subjective experiences of the children in their relationships and networks. We also considered their welfare and standard of living, which include livelihood and satisfaction with their lives and the human dimension, which includes their personal safety, emotional and mental health and wellbeing. Using this framework, we have shown the interaction and interdependence of the three dimensions—relational, material, and human in each space and at a given time in the lives of the children.

### The socio-demographic characteristics of mothers and children

The mothers in this study were between 29 and 46 years old. The majority were Christian, and at least 90% had attained some schooling that varied from a few years to advanced level education (attained by two mothers). Most mothers were single parents living with their children, and a few reported that they were taking care of orphans. The median number of children born to the mothers was four, with a range between two and six (see Table 1).

**Table 1:** Socio-demographic data for the mothers of the children enrolled in the study from the GHWP.

Characteristics of mothers	Overall	≤30 years	31–34 years	35–40 years	≥41 years	*p*-value
Age						
Median (IQR)	39 (35–43) years	30 (29–30) years	34 (33–34) years	38 (36–39) years	45 (43–46) years	0.0001*
Religion						
Catholic	25 (41.7%)	2 (8.0%)	6 (24.0%)	9 (36.0%)	8 (32.0%)	
Church of Uganda-Anglican						
Muslim	17 (28.3%)	2 (11.76%)	3 (17.65%)	7 (41.18%)	5 (29.41%)	
Born again	10 (16.3%)	0 (0%)	1 (10.0%)	4 (40.0%)	5 (50.0%)	
Overall	8 (13.3%)	1 (12.50%)	0 (0%)	7 (87.50%)	0 (0%)	
	60 (100%)	5 (8.33%)	10 (16.67%)	27 (45.0%)	18 (30.0%)	0.266
Level of education						
Never	3 (5%)	0 (0%)	1 (33.33%)	2 (66.67%)	0 (0%)	
Primary level	40 (66.7%)	2 (5.0%)	6 (15.0%)	16 (40.0%)	16 (40.0%)	
Ordinary level	15 (25%)	3 (20.0%)	2 (13.33%)	8 (53.33%)	2 (13.33%)	
Advanced level	2 (3.3%)	0 (0%)	1 (50.0%)	1 (50.0%)	0 (0%)	
Overall	60 (100%)	5 (8.33%)	10 (16.67%)	27 (45.0%)	18 (30.0%)	0.211
Marital status						
Married to a man alone	5 (8.3%)	0 (0%)	1 (20.0%)	4 (80.0%)	0 (0%)	
Married with a co-wife	5 (8.3%)	2 (40.0%)	2 (40.0%)	1 (20.0%)	0 (0%)	0.037*
Widowed	1 (18.3%)	1 (9.09%)	0 (0%)	4 (36.36%)	6 (54.55%)	
Separated/Divorced	39 (69%)	2 (5.13%)	7 (17.95%)	18 (46.1%)	12 (30.77%)	
Overall	60 (100%)	5 (8.33%)	10 (16.67%)	27 (45.0%)	18 (30.0%)	
Number live children						
Median (IQR)	4 (3–5)	2 (2–5)	4 (2–5)	4 (3–6)	5 (4–6)	0.322
Age of these live children						
Median (IQR)	15 (14–18)	14 (13–15)	14 (14–16)	15 (13–18)	17 (14–18)	0.0958
Take care of orphans						
Yes	36 (62.07%)	5 (13.89%)	3 (8.33%)	15 (41.67%)	13 (36.11%)	
No	22 (37.93%)	0 (0%)	7 (31.82%)	10 (45.45%)	5 (22.73%)	0.043*
Father providing support to children						
Yes	13 (21.67%)	2 (15.38%)	3 (23.08%)	8 (61.54%)	0 (0%)	
No	47 (78.33%)	3 (6.38%)	7 (14.89%)	19 (40.43%)	18 (38.30%)	0.019*

*N* = 60, Kampala and Wakiso District, Oct 2020-May 2021. Row % used within age groups, significant values are denoted by * indicating values *p* < 0.05 based on Fisher’s exact test.

The children in this study were aged between 12 and 23 years. There were fewer boys than the girls. Over half of the children were in school. Five of the children reported having experienced physical violence in the previous 3 months before the study (see Table 2).

**Table 2:** The socio-demographic data from the children of mothers attending the GHWP.

Socio-demographic characteristics of children	Overall	12–15 years	16–17 years	18–24+ years
Age	60 (100%)	31 (51.67%)	14 (23.33%)	15 (15%)
Median age (IQR)	15 (14–18) years	14 (13–14) years	16 (16–17) years	19 (18–20) years
Sex				
Male	24 (40%)	13 (41.94%)	4 (28.57%)	7 (46.67%)
Female	36 (60%)	18 (58.06%)	10 (71.43%)	8 (53.33%)
Overall	60 (100%)	31 (51.67%)	14 (23.33%)	15 (25%)
Currently in school				
Yes	46 (76.67%)	29 (63.04%)	10 (21.74%)	7 (15.22%)
No	14 (23.33%)	2 (14.29%)	4 (28.57%)	8 (57.14%)
Overall	60 (100%)	31 (51.67%)	14 (23.33%)	15 (25.00%)
Experienced domestic violence in last 3 months				
Yes	5 (8.33%)	3 (9.67%)	0 (0.0%)	2 (13.33%)
No	55 (91.67%)	28 (90.32%)	14 (100%)	13 (86.66%)
Overall	60 (100%)	31 (51.67%)	14 (23.33%)	15 (25.0%)

*N* = 60.

In the next section, we present the children’s experiences of vulnerability and resilience under the following themes: social relationships, financial wellbeing, and human wellbeing (quality of the environment, personal safety, and emotional health). We adopted and adapted aspects of the wellbeing framework to situate our findings from the children in this study. Some of the findings from the questionnaires are shared together with the complementary findings from the qualitative data under some of the subthemes.

The summary of the themes and subthemes developed and drawn from the patterns that emerged during the analysis of the qualitative data is provided in Table 3.

**Table 3:** Summary of themes and sub-themes.

Theme	Sub-themes
Social relationships	Children’s relationship with mothers and fathers
	Children’s relationship with other relatives
	Children’s relationship with peers
	Benefits and challenges of relationships
	Support systems
Material and financial wellbeing	Source of economic support
	Food security
	Schooling
Human dimension/environment	Environment: set up/living conditions in context
	Activities in community
	Personal safety
	Emotional/mental health (psychosocial)

### Social relationships

The social relations that the children identified with and how satisfied they were with these were analysed under this theme. The main social relations included relationships with their mother and father, with relatives, and with peers. The value attached to the relationships was similar across all the ages of the children. Under this theme, we highlighted the positive and negative experiences in relationships and how the children accessed social support networks in the community.

#### Relationship with mother and father

Of the 20 children who took part in the life-history interviews, 10 lived with their mothers, and 9 lived with grandmothers, usually on the maternal side; one child reported living with a grandfather. Fathers were usually absent, although in a few cases, they had died. In the life histories, two boys mentioned their concern that they had never seen their fathers and they wanted to know about their paternal relatives; however, their mothers would not, or could not, tell them who their fathers were.

Although most of the children did not have an experience living with their fathers and were usually bitter towards absent fathers, there were a few who appreciated their fathers’ support and involvement in their lives. One girl, aged 22, mentioned that her father was her main source of knowledge and told her about menstrual hygiene. To this girl, the father, who was not living with the mother, was the main source of resilience and support as she grew up.

Most children reported good relationships with their mothers, and for some, their mothers were their heroes. The girls aged 18 years and above who were interviewed during the life histories and the FGDs did appreciate the advice of their mothers, especially in relation to avoiding sex when they were young. A majority of the children *N* = 45 (75%) reported during the questionnaire interview that they were usually under the watch of their mothers or caretakers, so they could not get involved in activities such as taking drugs and going out with friends.

#### Challenges faced while relating to their mothers

The children reported that mothers gave different forms of punishment, but usually, it was a physical beating. During the questionnaire interview, five children reported having received physical punishment in the previous 30 days. Punishment included slapping (1), beating (4), kicking, and pushing; one child reported being dragged on the ground by the mother in addition to being beaten. Punishment varied by age group; for example, older children aged 18 years and above were usually counselled by their mothers and not beaten like younger ones when they made mistakes. The same information emerged during the FGDs and the life histories.

Parents were also accused of verbal abuse in the qualitative data:

*Some parents are vulgar [use vulgar language] in front of children. They raise children, telling them that they are stupid… The child can end up also speaking obscene words to people.* (Boy, 12–17 years, FGD)

Three children over 18 years old reported that they felt treated differently from other children at some point in their lives by the mother. The main reason was that the mother’s current partner was not their biological father.

#### Relationship with other relatives

Children had no choice when it came to where they lived; this depended on the circumstances of the mother. To most children grandparents played a key role in their lives while growing up.

The duration of stay with the grandparents varied from infancy to adolescence. One girl said:

*I grew up believing that my grandmother was my mother, and even when my mother used to come and pay a visit to us, I used to tell her to go back to her home*. (Girl, 23 years, life history)

An 18-year-old boy mentioned he had stayed with his grandparents because his mother was in a new relationship, which was painful to him:

*I have spent eight years at my grandparent’s home, and I came when I was still young. We came straight here to my grandparents’ home, and mother was still looking for money to build a house because she had gotten another partner, and we should not stay with him there, so she brought us here*. (Boy, 18 years, life history)

Children were usually saddened by the fact that they had to live away from their parents, and interestingly, the boys voiced this more emphatically than the girls:

*I left my mother though I still wanted to stay with her, but they saw that she was grooming me in a rather relaxed way because I am the only child she has. So, when she took me to my grandmother’s place, they told her to leave me there, and I began staying at my grandmother’s home when I was between 3 and 4 years old*. (Boy, 18 years, life history)

Some girls reported their struggles living with relatives and stepfathers because these adults were, at times, not friendly and had sexual demands that put them at risk of getting pregnant. One 14-year-old girl, during her life history interview, reported abuse from a relative because they were sleeping in the same room; it stopped after she reported it to her mother:

*Well, … when he woke me up, he would touch me for a moment, and I said to him, “leave me alone”, and he said, “You wake up,” and I reported him that very day, and he was blamed and he stopped*. (Girl, 14 years, life history)

Our findings from questionnaires, FGDs, and life history interviews showed that although children were living with their mothers, and many were happy about their relationship, some reported challenges in their relationship with their parents and some close family members. Twelve children reported during the questionnaire interviews that they had been insulted, humiliated, or called names by parents (2), relatives (1), peers (4) and unnamed others (5). Two boys, one aged 15 and the other aged 20 reported during the questionnaire interview that they had been denied food in the past 30 days. Two children said they had been denied shelter in the past 30 days—one, a boy aged 14, and a girl aged 16.

#### Relationship with peers

Most children lived and grew up in communities where they were able to interact with peers, and some had positive experiences, such as working together with peers to get income. However, some children, especially boys, reported negative influences from some of their peers, which included stealing people’s property and alcohol and drug use. During the FGDs, three boys (aged 15–18) reported having taken alcohol at some point due to peer pressure.

One girl, aged 17, confided that she had been touched in the genitals by peers; she had not reported it to anyone. Some girls reported being put in vulnerable situations by their friends. One 19-year-old girl reported having been asked by a friend to stay in her house while she was away. The partner of the friend came back and wanted to have sex with her:

*This is a secret, though I informed my mother. My friend was staying at L and had a partner. She requested me to go and stay at her house, and she left me there with her partner. As you know men, he came back drunk and came straight to my bed, and I was sleeping, and he told me that he was in love with me*. (Girl, 19 years, life history)

Some girls disclosed during the FGDs that they were invited to work with their peers to manage harsh conditions while living with relatives or working as domestic help. One 22-year-old girl during the life-history interview reported peer influence that led to trying out sex work a year before the interview. She reported that she later abandoned sex work because she was being forced to have sex with men without using a condom.

*It was a short time, about a month, and I abandoned it; I used to find it hard. Occasionally, you could get someone who would refuse to wear a condom, so I left it and when I tested myself for HIV and found that I was OK [not infected]. I abandoned it, and I will not return to it*. (Girl, 22 years, life history)

During the FGDs, the children mentioned communal activities that drew them close to each other. For the girls, the main bonding activity included community dancing, which was encouraged to provide skills to girls so they could earn some income. However, most girls before the age of 20 were not allowed to move about in the community by their mothers or caretakers, worried about them getting pregnant. The boys were less restricted, and they were usually free; even a 12-year-old could move about freely and watch or play football.

#### Benefits from relating with others

Despite the hard circumstances that were reported by the children in different relationships, many reported coping with it by putting their trust in God and becoming attached to the mother or grandparent who cared for them.

One boy was supported by friends when he was rejected by a stepmother and told to leave home. He reported that his peers introduced him to collecting plastic bottles from garbage and selling them to earn a living. We share a short excerpt from the interview:

INT: *So, when she [stepmother] chased you, where did you go on that day?*

RES: *I slept in an unfinished house. I had friends who were collecting rubbish, that is when I last went to school. We lived in these houses [incomplete] with doors, but when people have not entered them yet. You lay down on a polythene bag and sleep.* (Boy, 18 years, life history)

#### Support services and social systems in the community

The children, while responding to the questionnaire interviews, named the following social services in their communities: churches 60 (100%), mosques 60 (100%), schools 51 (85%), and the police 52 (86.7%).

During the FGDs, the children reported services in the community and individuals from some organisations who sometimes offered HIV outreach and counselling to young people. Three children reported having received school sponsorships. Most children reported being encouraged and sometimes forced to go to a place of worship for prayer as most mothers felt the children would listen to priests, learn something, and receive counselling.

*There are churches and mosques which have helped a lot to enable people go through difficult problems. You may go and talk to the pastor, priest, or sheikh and tell him your problem, and they take your message to other people who collect money for you, and they give you that help*. (Boy,18–24 years, FGD).

#### Support systems in the community

During FGDs and life history interviews, children acknowledged the presence of support systems, including neighbours who came to their rescue when needed. These varied from friends, peers, and relatives, and some children reported getting support from community social services like the church. An example that was reported during a life-history interview by a 12-year-old boy:

*By the time I began to understand, I had friends called “D,” “O,” and “D” and they were Somalis. They were very good friends of ours, our mother used not to spend the day at home, and we used to spend the day with “D” seeing films. They used to give us lunch and supper, and we used to eat with them*. (Boy, 12 years, life history)

Sometimes, children defied the mothers’ and neighbours’ rules put in place to control children’s negative behaviour.

*They stopped us from roaming around, but we do not obey instructions. So now mother puts spies so that if we move around, her spy immediately phones her even if it is a short movement, say like going to the toilet; I have to inform the neighbour, and she understands it. If you do not inform her and she sees you coming back, she will immediately phone your mother and report you and mother will come and beat you in anger*. (Girl, 14 years, life history)

Although some community support systems were good, during the FGDs, the children shared a few negative experiences, as reported by one young girl.

*Some of them I call bad; they are drunkards, and instead of counselling you, he gives you money with the intention of making you his sexual partner (wife)* (Girl, 12–17 years, FGDs).

The social relationship dimension of the children, according to our findings, has many facets, and it is in many ways intertwined with the human and material dimensions. The role of grandmothers on the maternal side and grandfathers on the paternal side were a source of support when mothers did not have enough support for their children.

### Human dimension

This dimension of the wellbeing framework is concerned with participants’ capabilities, attitudes to life, as well as personal relationships. In this theme, we explored the children’s experiences on how they gauged their environment, what activities went on and how they affected them, household structures, and their views on their mental and emotional wellbeing.

#### Quality of the environment

The children’s living conditions were diverse; whereas a few noted that they were in clean neighbourhoods, the majority said they lived in filthy surroundings.

They reported behaviour such as stealing from neighbours and risking violence or being taken to prison for both girls and boys because of alcohol and drug use. The children reported frequent witnessing of domestic and physical violence by parents and relatives in the communities where they lived. All the children in the life histories and FGDs mentioned alcohol and drug use in their communities. This was reported as leading to violence in homes, some people being raped and children defiled, children and mothers being beaten; a few young people had become mentally unwell due to drug use.

*You may find someone on the road behaving as a mad man, yet he used to be a well-behaved person. This person may have been taught to smoke opium by one of his colleagues, and little by little, he caught the habit until he smoked too much of it, and it made him lose control of his mental health*. (Girl, 14 years, life history)

Fighting between husbands and wives was common in the communities, and children reported its influence on families becoming separated.

*People fight each other. There was a man who got drunk, and when he returned home, he quarrelled with his wife and struck her on the head with a bottle due to alcohol.* (Girl, 23 years, life history)

Not only were disputes between parents common, but the effects of alcohol abuse often affected children.

*I had a friend, and at her home, her father used to drink alcohol. Whenever he did so, he would beat them, beat their mother, and kick the TV to pieces, and they used to buy a new TV every month*. (Girl, 19 years, life history)

According to the children, access to drugs and alcohol was easy, especially when sellers would provide small portions affordable for anyone.

*There are some shops which sell kuba [a drug]. They get hold of the black polythene bag which they tear in pieces and start packing in small amounts and display it there. Whoever wants but does not have 2000 (Uganda shillings, equivalent to USD 0.75) [for the bigger package] can buy for 500 (Uganda shillings, equivalent to USD 0.15).* (Girl, 17 years, life history)

During the questionnaire interview, 24 (40%) children stated that they did not have enough privacy while bathing at home, which was a high risk for being sexually abused even by close relatives, such as uncles or neighbours. The living conditions were reported to be poor, especially because the majority lived in single rooms with parents and relatives.

*The house is not spacious enough. When I am inside at night, I cannot breathe well. We have only one window in the house, and there is no way I can open it because it touches the house of neighbours.* (Boy, 12 years, life history).

As many as 17 (28%) children reported during the questionnaire interview of being exposed to adult content, including pornography, through social media using their mothers’ phones or through television programmes that, in their view, were not appropriate.

#### Belonging and personal safety

During the questionnaire interview, the children were asked whether they had knowledge of their rights. They were asked through structured questions to respond to what they knew as their rights. All the children (60) were aware that they had rights; however, when asked, these were not very clear to them. The rights identified included the right to express views as children (28), the right to a name (3), the right to life (14) and the right to protection from economic and sexual exploitation (22). Most of this knowledge had been acquired from schools.

Despite their knowledge of key aspects of their rights, this was not always realised in their context. Some children noted that they had to live with stepmothers, stepfathers, grandparents, and uncles, who, on multiple occasions, put their lives in very vulnerable situations. The lack of privacy was a challenge to several young people, especially girls.

*You know women have menstrual periods, and the place at home is small. When I go to the bathroom outside and bathe, I should come out dressed having done the necessary activities. But before you can finish those activities, he [stepfather] complains that I have spent a long time in the bathroom and that I should come out and let him bathe. So, I move out and go to the neighbours’ bathroom*. (Girl, 17 years, life history).

#### Emotional health and wellbeing

Children were asked to rate their mental wellbeing as “never,” “a little,” “sometimes,” or “most of the time” combined using an anxiety and depression checklist. Our findings showed that most children had not experienced anxiety; however, some had gone through difficult times. Some children reported feeling hopeless, tired, nervous, worthless, and sad (Tables 4 5).

**Table 4:** Self-reported assessment level of anxiety and depression by children aged 12–23 years.

Mental health (anxiety and depression)	N (%)	Odd (95% CI)	*p*-value
Often felt tired	26 (43.3%)	0.76 (0.46–1.27)	0.303
Feel hopeless	21 (35.0%)	0.54 (0.32–0.92)	0.022*
Feel nervous	19 (31.7%)	0.46 (0.27–0.79)	0.006*
Feel sad	20 (33.3%)	0.5 (0.29–0.86)	0.011*
Feel worthless	4 (6.7%)	0.07 (03–0.19)	0.000*
Feel restless	4 (6.7%)	0.07 (03–0.19)	0.000*

*denotes the following: 0.000 very low, 0.11not significant, 0.006 statistically significant, 0.022 significant.

**Table 5:** Comparison of crude odds ratio and adjusted odds for age for anxiety and depression.

Mental health (anxiety and depression)	Odd (95% CI) unadjusted	Odd (95% CI) adjusted (12–15 years)	Odd (95%CI) adjusted (16–17 years)	Odd (95% CI) adjusted (18 years+)
Often felt tired	0.76 (0.46–1.27)	Ref	2.4 (0.67–8.79)	1.6 (0.45–5.57)
Feel hopeless	0.54 (0.32–0.92)	Ref	0.97 (0.24–3.94)	2.8 (0.78–10.0)
Feel nervous	0.46 (0.27–0.79)	Ref	5.2 (1.23–21.5)	4.6 (1.13–18.4)
Feel sad	0.5 (0.29–0.86)	Ref	1.8 (0.49–6.8)	1.2 (0.33–4.6)
Feel worthless	0.07 (03–0.19)	Ref	1	2.2 (0.28–17.6)
Feel restless	0.07 (03–0.19)	Ref	1	2.2 (0.28–17.6)

The findings under this theme on the human aspects of the children in relation to their experiences and attitudes to life, their mental wellbeing and the context and structural aspects that affect them reveal some negative experiences but also show that the children challenge the status quo by demonstrating resilience and survival.

### Material/Financial wellbeing

This theme is linked to the material or financial wellbeing in the framework. This dimension emphasises the practical welfare and standard of living that may include sources of income, consumption and how satisfied the children are with their conditions. Under this theme, we explored what the children expressed in relation to income and their livelihoods.

The financial wellbeing of the child depended on the caretaker, who was usually the mother or grandparent. The quantitative and qualitative data showed that children faced challenges related to material and financial wellbeing. The major challenges included insufficient food, lack of school fees, and the insecurity that surrounds living in crowded rental places surrounded by many bars in the neighbourhood.

#### Food and nutrition

In the questionnaire interview, most of the children (*N* = 51, 85%) reported that they had two to three meals a day, 8 (13.33%) had only one meal a day, and one child reported at least four meals a day. The mothers’ data (not shared in detail in this paper) showed that most of them (57) had to buy food from the market to be able to feed their children. A 22-year-old girl said: we *can fail to get what to eat. There is a day you can wake up and you do not know what you are going to eat, and all of you end up looking at mother* (Laughs).

#### Education

The uncertainty of schooling was felt by most children, especially because of irregular attendance.

*My father died when I was heading to P.5, but before he died, I was living with my mother, and when he died, I was taken by my cousins, though they didn’t treat me well. My teachers took me back to my mother’s place, and mother took me to a friend’s place where I would live and go to school. They were not valuing education as I would go to school twice a week because I would be engaged doing house chores before going to school*. (Girl, 12–17 years, FGDs)

This kind of situation was common in most of the children’s lives, with frequent changing of schools as a result of a lack of school fees and logistics.

#### Housing and accommodation

Children were disadvantaged because of having to move from one place to another or from one relative’s home to another because of the failure of mothers to pay rent.

*We moved, and we spent there four months, and we left and came here, but we are also going to move away from here because they are raising the rent after a short period, and mother cannot pay the rent alone.* (Girl, 14 years, life history)

The safety and wellbeing of the children in the community were influenced by available work, including small businesses of restaurants, garages, saloons, and roadside businesses. These jobs were sometimes also done by children. The children reported that sex work was one of the jobs that people in their communities engaged in.

*There is sex work in the area and garbage collection. There are other youth who collect garbage; he collects garbage from your home, you give him money and he finds somewhere to dump the rubbish*. (Boy, 12–17 years, FGDs)

One 15-year-old girl in a life-history interview reported that she suspected her mother did sex work for a living. When she asked her mother why she spent nights out of home, she was beaten and never asked her mother again. However, she was saddened by what her mother was doing. She recollected: *I do not have anything that has made me happy in life*.

Another 16-year-old girl disclosed that she started to do sex work at some point to earn some income but stopped because it was a very difficult job.

### Aspirations and resilience

Based on their life histories, it was apparent that all the children aspired to become someone important in their community. Their dreams included becoming health workers, nurses, doctors, car mechanics, and lawyers. Some even dreamed of setting up big businesses, including beauty parlours, boutiques, restaurants, and shops. A 13-year-old boy shared: *In future, I would like to have a job being employed as a mechanic.*

One young girl wanted to become a health worker.

*I would like to be a nurse. I want to treat people and they get cured because if you are a health worker, you can even treat peoples’ minds by counselling them and save them from difficult situations.* (Girl, 15 years, life history)

One of the children who dropped out of school still harbored hopes of becoming a lawyer. Most children, despite the challenging context, had trust and hope in God and their mothers and believed that they would cope with what happened in the future. One girl remarked:

*God has been the main reason why I have been able to reach this far. Without God, nothing is possible, and the other person is my mother because the situation we are living in pushes me to be a hardworking woman, and I am proud of that because I will not wait on people to give me.* (Girl, 22 years, life history)

## Discussion

In this study, we explored the lived experiences of children of mothers who earn income by selling sex for money and goods. We collected qualitative and quantitative data to triangulate findings from interviews with deeper stories from life histories and FGDs. Our findings revealed some concerning realities that children born to mothers involved in sex work experience as they grow. We used a wellbeing framework for development practice to analyse and contextualise the data.

Our study revealed that the lived experiences of children can be detrimental to their social, human, material, and mental health wellbeing. Their social relationships and interactions with family members, relatives, peers, and other community members, as well as their financial wellbeing and mental health, were all subject to what was happening in their context at any given time.

Mothers were seen as the closest persons in the lives of most of the children, with the limited presence of father figures for most. Anxiety experienced by some children reveals a need to explore the context to treat and prevent mental illness among these children. Despite being surrounded by different and distinct challenges, many children were resilient and were able to face these hardships; they believed that the following day and years would be better.

The socio-demographic data of the mothers confirmed that there were many single mothers with an average of four children. This meant sharing limited resources and challenges in discipline, which at times ended up being harsh. Negative influence from peers and environmental and structural limitations exposed some children to vulnerable situations. Some of the women were also responsible for orphans of family members—a common support network received from relatives.

More than half of the children reported being in school, even though they were not able to attend classes consistently, which is the current requirement by the Uganda government. According to the national demographic and health survey, at least 86% of children aged 6–12 attend school (16); however, in the context of the children in this study, peculiar challenges exist that affect regular school attendance, and most of them are outside the official age range for primary school. Of the 14 children who were not in school, 6 were under the age of 18, which is a challenge for their future development and economic opportunities.

Our findings also surfaced significant physical violence perpetrated by the mothers on their children. This requires attention and appropriate intervention. A UNICEF survey revealed that most physical violence that was reported by children aged 18–24 years began at or before age 5 in Uganda; unfortunately, parents were some of the perpetrators named (17). A study in Chile has reported on the tremendous impact violence in family and communities have on youth; these may result in them becoming aggressive (18).

Structural factors such as access to schools, medical care, and social services in the children’s settings also contributed to their wellbeing or vulnerability at different points in time. While mobile phones could be a positive tool to acquire knowledge, we also noted that children watched what they admitted was inappropriate, similar to a study conducted in Kampala among young sex workers aged 14–23 years who accessed inappropriate content on phones even with limited internet access (19).

Several children showed resilience in their situations, which was facilitated by trust in their mothers, but we had some children who also reported varying levels of anxiety and depression, which calls for concern. Those experiencing anxiety are an important group that may reflect silent pain and may require more structural support systems, including counselling services for young people in every community. A recent scoping review showed that mental health practitioners can boost resilience in children and adolescents by addressing the relational and personal support mechanisms embedded in African contexts, which emphasises ways of doing and being (20).

Our study shows that social structure networks are still important in present-day societies despite the changes that have been brought about by technology; physical interaction with family members is key in cementing relationships with the family and the community. However, physical and verbal abuse by relatives and peers is an aspect that needs to be dealt with to reduce children’s exposure to physical and emotional violence. A national survey revealed that 1 in 3 girls in Uganda suffer sexual violence during their childhood, and 7 in 10 boys face physical violence. This needs to be addressed (21). Mothers at high risk of HIV and engaged in sex work have admitted to remaining in the trade for the sake of supporting their children, and all the children’s voices indicated the importance of the mother (6, 7).

A recent study conducted in the United Kingdom assessed aspects of bonding between mothers who do sex work and their children. The study reported a need for support systems in the community to uphold both mother and child (22). The challenge that was reported about mothers working at night and often leaving children on their own is disturbing to the children and, in some instances, has created a risk to the children’s personal safety. A peer support system may be useful in this kind of environment. A recent study showed the importance of peer support for adolescents and young adults, which may help reduce anxiety by enhancing access to required or shared resources (23). However, another study on peer influences on substance use revealed that there could be different levels of influence on the children in the control of drug use, such as the effect from a close friend or lack of social support from mothers (24). This is an area to explore and increase social interactions in the community so that no adolescent or young child is left on their own without a social support system.

Peers were seen to be part of the support system when there was a crisis and sometimes a source of resilience, especially for boys. A study in the US looking at positive and negative influences that may accelerate crime showed that influences from mothers or siblings during childhood were generally positive (25).

The social environment, especially where there is alcohol, was a challenge because it left both girls and boys in a very vulnerable situation. For girls, the temptation to get support from older men and for boys, the struggle to survive financially, along with some frustrations, has led to alcohol abuse and drug addiction—although few boys reported drinking alcohol in this study. Several studies have shown that alcohol use and abuse by parents can lead to poor quality of parenting and easily cause young adolescents to drink alcohol and use drugs (26, 27).

The financial situation of these children left many of them in a helpless situation of lack, insufficient food, lack of accommodation, and irregular schooling; these hinder development and may easily lead to a cycle of mother and child becoming involved in sex work for money and goods.

Structural factors such as the lack of reasonably sized accommodation due to financial constraints affected personal safety and was a threat to both boys and girls in this study (28). A study conducted among young people in relation to PrEP (pre-exposure prophylaxis) uptake revealed that the cost of PrEP was a barrier to access (29).

Our findings revealed that although children may find it difficult to express their hurt or pain to adults, these challenges still exist. A child who reports being sad and hopeless is a source of worry to the community because they may end up taking drugs, have suicidal tendencies, or may cause harm to others (30). These findings point to the need for counselling services targeting children and adolescents. The reported prevalence of domestic violence is a sign of concern in relation to men’s and women’s rights. Although the children reported knowledge of rights, there are likely reasons for not reporting perpetrators, such as the need for financial support and trying to avoid the breakdown of social networks in the community. Research on violence shows that more in-depth follow-up of victims and involvement of different stakeholders are required to manage this challenge (31).

Situations such as the one disclosed by a girl who was almost raped by a friend’s husband and only managed to escape because the mother had shared some tips on self-defence are important for young people who live in environments that are at high risk of gender-based violence (GBV). A systematic review conducted to explore the effectiveness of GBV interventions on young people living with or affected by HIV in low- and middle-income countries (LMICs) showed that social health, empowerment, self-defence, and gender sensitisation interventions might be effective for GBV exposure, although they may not be able to deal with perpetrators (32). The other factor that young people kept referring to was their belief in God. We propose one way forward would be first to strengthen the social support networks at the community level, which would include supporting socio-economic enhancement in less-privileged communities, specifically with women who offer sex for money and goods. Structural factors such as a lack of assured medical care for adolescents living in vulnerable environments will continue to lead to inequalities in society unless tackled through policy change.

## Limitations of the study

The children in this study were chosen by their mothers. Therefore, we cannot be sure that they were the best representatives of the families among this cohort of women. The findings, however, offer real-life experiences voiced by children, and these findings can lead to interventions among this population. The study targeted a few children, given that this was a pilot descriptive study to inform larger studies.

## Conclusion

Children of mothers at high risk of HIV who sell sex for money or commodities experience structural, contextual, and mental health challenges. They desire to survive and succeed despite the hardships they face, which demonstrates resilience. Interventions that comprehensively target the child from 5 years to adolescence should be designed and integrated into community social services. Services and interventions should include socio-cultural, economic, health, and psychosocial support for children in these low resource urban areas. There is a need to strengthen communal support systems for children living in similar circumstances in low- and middle-income countries.

## Data availability statement

The raw anonymised quantiative data supporting the conclusions of the article can be made available by the authors, without undue reservation. Requests to access qualitative data will be given consideration by the authors,in accordance with confidentiality agreements signed with participants for the use of their data.

## Ethics statement

The studies involving humans were approved by the Uganda Virus Research Institute REC. The studies were conducted in accordance with the local legislation and institutional requirements. Written informed consent for participation in this study was provided by the participants’ legal guardians/next of kin.

## Author contributions

AS: conceptualisation and writing original draft. AS, GN, and IS: data collection. AS and MM: formal analysis. AS, JS, and RK: funding acquisition. All authors contributed to the article and approved the submitted version.

## Footnotes

^1^https://www.beyondblue.org.au/the-facts/anxiety-and-depression-checklist-k10
