# Supplementary material for: Contextual, structural, and mental health experiences of children of women engaged in high-risk sexual behaviour in Kampala: a mixed method study
Source: Front Public Health. 2023 Dec 15;11:1185339. doi: 10.3389/fpubh.2023.1185339 (PMC10773752; doi:10.3389/fpubh.2023.1185339)
Supplement: Supplementary file 1 [file Data_Sheet_1.docx]

**Focus Group discussion guide- WHR and children study**

**Introduction**

Moderator and co-moderator/note taker receive participants at the agreed upon venue.

Introduction by moderator and note taker

Introduction by each participant – name, and any other thing of interest about them

Explanation of study and aims for the FGDs

*The study aims to understand better how children of mothers attending the Good health for women project clinic grow up, what experiences mothers go through as they bring up their children and what experiences children go through as they grow up. The study aims to identify the socio-economic and health context that the children of high risk women grow up in and to identify areas for possible interventions that are low cost and fit in the culture of the mothers and their children.*

Set ground rules – each participant is important and there are no wrong answers because we are talking about experiences in the community, wait for each other to complete idea before we speak out our idea

Moderator introduce recorder and get verbal consent from each of the participants to use the recorder.

**Topic 1 Life in the community**

What kind of work goes on in the community to earn income? (Probe for all work, formal and informal jobs and occupation in the community

**Topic 2 Bringing up children**

How are children brought up in this community (Probe about is responsible for child upbringing, common habits, disciplining children)

What are the expected behaviour of children as they grow up in homes and then in community-ask about the ones aged 12-15 years, 16-17 years, 18 plus years)? (Probe for any behaviours, such as eating, respect, relating with adults and aspects about their culture and social norms that are discussed spontaneously by the respondent)

What are the social networks responsible for children growing up in the community? Who are responsible for seeing that children grow up well, in acceptable cultural norms? Get the group to discuss the social and cultural norms? (Probe for those that may be positive and negative, and ask what has changed if anything)

**Topic 3 Socialisation**

How does socialisation happen in this community, who initiates the process and why?

Culturally who is responsible for helping children socialise?

Socialising process for girls and boys (how is this handled and by who in this community?

Probe about sexual behaviour in the community (norms, gender roles)

How do people cope with difficulties and challenges in this community (loss of close family member, child, parent, loss of income, sickness/chronic disease?)

What strategies are there in the community to support individuals, children and youth and families in the community to cope with the situations you have raised and how are you able to keep going?

**Topic 4 Alcohol and substance use**

Let us talk about alcohol use in the community (Probe about number of bars, lodges, rules about end time and begin time). Any instances of alcohol misuse? What could be the solutions to this?

What kind of violence occurs in this community towards children, women and men-ask for each group and what type(s) of violence occur in the community (physical, child beating, sexual violence)?

Ask about factors that affect general health wellbeing of children in the community

**Topic 5 support systems/networks**

What economic support systems are there in the community for men, women, children, youth when there is need and or crisis in a family?

What education support systems are there in the community for children and adult learners?

What social support systems are there in the community for men, women, children and youth when there is need in a family?

What health support networks exist in the community to support individuals, children and youth in the community?

What youth friendly health and social services are available in this(your) community?

**Thank the participants**, inform them that when we are done with looking at all information collected, we will disseminate findings in seven or eight months’ time.
