# Supplementary material for: Contextual, structural, and mental health experiences of children of women engaged in high-risk sexual behaviour in Kampala: a mixed method study
Source: Front Public Health. 2023 Dec 15;11:1185339. doi: 10.3389/fpubh.2023.1185339 (PMC10773752; doi:10.3389/fpubh.2023.1185339)
Supplement: Supplementary file 2 [file Data_Sheet_2.docx]

**Life history guide for children- WHR study version 1.1**

***Interviewer- Welcome the child/ young person in a safe environment. If possible, try to avoid inside a small house and in a place where others will be listening in and you cannot record well.***

**Note**

**Age**

**Sex**

**Community**

Tell me about your life from the time you were born and could understand what is going on around you and in your family.

**Probe** about schooling, social life at home, behaviours common in home, habits in home, discipline in home, relationship with mother and or father if present, siblings, ask about friends/peers.

What have been the things you have liked most as you grew up and why

What have been the difficult situations that you have gone through in your life?

How have you been able to overcome the difficult situations?

What keeps you going even when there are difficulties or challenges? (Probe about people, activities, things etc)

Ask about economic situation in home (**Probe** about provision of food, schooling, clothing, and other basic necessities like shelter)

**Probe** about hopes and desires for the future and anticipated ways of getting to the desired end/future

Ask about current state of health, **Probe** where they go to get treatment when sick

Ask about HIV (**Probe** for transmission, prevention, fears, hope)

Ask about safety:

including if they have experienced any violence such as sexual abuse, probe by whom, whether it was reported and to whom, and what was done to perpetrator) or other violence

including any other reasons to feel unsafe (have there been floods or fires that made participant feel unsafe)

Ask about alcohol use, substance use (**Probe** for individual involvement, family). Ask about challenges experienced in family due to alcohol misuse or drug substance

Ask about youth programs in the community (**Probe** for services, sports, /games, socialising events and any that the respondent is able to share)

What are the strategies in your view that can lead to improvement in your family life and community (handle each alone- family, then community)?

**Probe** for health, economic stand, social network (ask for each in relation to family and community) example what are the health aspects that you think may lead to improvement in your family? What about in the community? What are the economic aspects that you think may lead to improvement in your family? What bout in community? What social networks are important for your family?

What have been the reasons you have managed this far in your life? (Reflect on his or her story and probe about social, health and economic aspects)

***Inform participant you have come to the end and allow her/him to ask any questions that they have***

***Thank the participant and give reimbursement for time spent with you.***
